# Transcriptome analysis of *Streptococcus pneumoniae* treated with the designed antimicrobial peptides, DM3

**DOI:** 10.1038/srep26828

**Published:** 2016-05-26

**Authors:** Cheng-Foh Le, Ranganath Gudimella, Rozaimi Razali, Rishya Manikam, Shamala Devi Sekaran

**Affiliations:** 1 Department of Medical Microbiology, Faculty of Medicine, University of Malaya, Kuala Lumpur, Malaysia; 2 School of Pharmacy, Faculty of Science, University of Nottingham Malaysia Campus, Semenyih, Selangor, Malaysia; 3 Sengenics Sdn Bhd, High Impact Research Building, University of Malaya, 50603, Kuala Lumpur, Malaysia; 4 Department of Trauma and Emergency Medicine, University Malaya Medical Centre, 50603 Kuala Lumpur, Malaysia

## Abstract

In our previous studies, we generated a short 13 amino acid antimicrobial peptide (AMP), DM3, showing potent antipneumococcal activity *in vitro* and *in vivo*. Here we analyse the underlying mechanisms of action using Next-Generation transcriptome sequencing of penicillin (PEN)-resistant and PEN-susceptible pneumococci treated with DM3, PEN, and combination of DM3 and PEN (DM3PEN). DM3 induced differential expression in cell wall and cell membrane structural and transmembrane processes. Notably, DM3 altered the expression of competence-induction pathways by upregulating CelA, CelB, and CglA while downregulating Ccs16, ComF, and Ccs4 proteins. Capsular polysaccharide subunits were downregulated in DM3-treated cells, however, it was upregulated in PEN- and DM3PEN-treated groups. Additionally, DM3 altered the amino acids biosynthesis pathways, particularly targeting ribosomal rRNA subunits. Downregulation of cationic AMPs resistance pathway suggests that DM3 treatment could autoenhance pneumococci susceptibility to DM3. Gene enrichment analysis showed that unlike PEN and DM3PEN, DM3 treatment exerted no effect on DNA-binding RNA polymerase activity but observed downregulation of RpoD and RNA polymerase sigma factor. In contrast to DM3, DM3PEN altered the regulation of multiple purine/pyrimidine biosynthesis and metabolic pathways. Future studies based on *in vitro* experiments are proposed to investigate the key pathways leading to pneumococcal cell death caused by DM3.

*Streptococcus pneumoniae* represents one of the major bacterial pathogens heavily affecting human health worldwide causing severe life-threatening infections particularly pneumonia, meningitis, and bacteremia^1^,^2^. Pneumococcal disease is the leading cause of vaccine-preventable deaths among children aged less than five with 0.7–1 million cases every year worldwide^3^,^4^. Treatment options are further reduced by the increasingly prevalent antibiotic-resistant *S. pneumoniae* particularly the multidrug-resistant strains in infections, inversely affecting the mortality and morbidity of patients^5^,^6^,^7^,^8^. Continued reduction in conventional antibiotic efficiency is inevitable and development of new classes of antibiotics as alternative antimicrobial agents is highly demanded.

Antimicrobial Peptides (AMPs) are characterized by short chain length (5–50 amino acids), polycationic, and amphipathic produced naturally by various organisms as effector defence molecules against bacteria, fungi, viruses, eukaryotic parasites, and others^9^,^10^,^11^,^12^. In line with new AMPs discovery from natural sources, researchers have been actively developing engineered AMPs with enhanced antimicrobial and reduced cytotoxicity as potential antibiotic candidates^13^,^14^,^15^,^16^. AMPs induced strong non-receptor mediated membrane lytic mechanism as the primary microbicidal strategy^17^,^18^. Three principal membrane disruption machineries have been described^19^. Toroidal pore (e.g. lacticin Q)^20^, barrel-stave (e.g. Alamethicin)^21^ and carpet models (e.g. cecropin P1)^22^, Aggregation of peptide monomers to form transmembrane channels or insertion of the peptides into the cell membrane to disrupt the native integrity of cell membrane eventually lead to direct cellular leakage and cell death.

AMPs possessing non-membrane targeting activity have also been increasingly documented^19^,^23^,^24^. Indolicidin, a Trp-rich polycationic peptide belongs to the cathelicidin family of polypeptides interacts with bacterial nucleic acids to interfere with cell replication or transcriptional processes leading to cell death^25^. Buforin II derived from the parent peptide buforin I inhibited cellular functions by binding exclusively to DNA and RNA without disturbing membrane integrity^26^. Histatin-5 is a mitochondrion inhibitor causing loss of transmembrane potential and generates reactive oxygen species which damages the cells^27^,^28^. Altogether, this indicates that the intracellular acting AMPs are able to traverse across cell wall and cell membrane efficiently and bind to the targeted macromolecules to exert inhibitory effects. Besides, peptides with multiple inhibitory effects have also been reported. CP10A, an indolicidin derivative was able to induce membrane lysis and inhibit DNA, RNA, and protein synthesis simultaneously^29^. PR-39 is another class of AMP interrupts with both protein and DNA synthesis pathways leading to metabolic cessation^30^. In addition, AMPs could produce varying inhibitory effects at different concentration. Lethal dose of pleurocidin would produce similar antimicrobial effects as CP10A as mentioned above, however, at sublethal dose the peptide was able to only inhibit protein synthesis by reducing histidine, uridine, and thymidine incorporations in *E. coli*^31^.

Advancement in Next Generation Sequencing platform for transcriptome analysis enables genome-wide expression studies on the cellular components and pathways affected by drug treatments via differential gene expression profiling. This includes previously known genes and novel expression systems, for example, the finding of two novel putative ABC transporters in *Streptomyces coelicolor* A3 (2) strain treated with vancomycin, bacitracin, and moenomycin A^32^. Qin *et al.* employed RNA sequencing (RNA-seq) to study the biofilm-inhibition potential of ursolic acid and resveratrol in methicillin-resistant *Staphylococcus aureus* (MRSA)^33^. Furthermore, specific gene expression can be identified by comparative analysis. For instance, the glyoxylate-bypass genes of the citrate cycle was upregulated in ampicillin-treated *Acinetobacter oleivorans* DR1 strain while norfloxacin induced significant SOS response^34^.

Our previous work had designed DM3, a water-soluble 13 amino acids cationic AMP generated based on hybridization of lead peptide fragments selected from the indolicidin-derivative peptide CP10A^35^ and the antibacterial peptide aurein 1.2^36^. DM3 showed potent antipneumococcal activity against both PEN-susceptible and nonsusceptible clinical isolates with greater killing kinetics as compared to PEN. In addition, DM3 is broad spectrum against common bacterial pathogens of both gram types. Combination with PEN synergized the antipneumococcal effect *in vitro*. Interestingly, DM3-PEN synergism was able to be translated into therapeutic improvement as shown in a lethal pneumococcal infection model using the non-toxic dose of the pair. Although the cell wall and cell membrane disruption potential of DM3 was evident, however, the detailed antipneumococcal actions of DM3 remain largely unclear. Here we aim at investigating the mechanisms of actions of DM3 in standalone and in synergistic formulation with PEN against *S. pneumoniae* via differential gene expression analysis using the high-throughput Illumina RNA-seq platform to identify the differentially expressed genes and the pathways involved.

## Results

### Transcriptomic analysis of PRSP and PSSP treated with standalone DM3 and in combination with PEN

**Figure 1:**
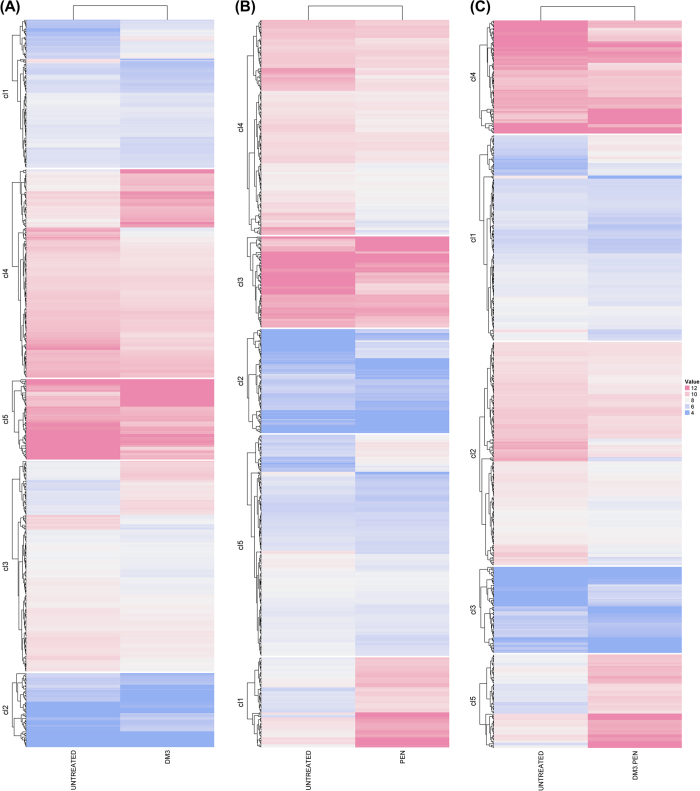
Heatmaps showing expression level of clustered genes of PRSP. Each group is classified into five clusters. (**A**) untreated versus DM3, (**B**) untreated versus PEN, and (**C**) untreated versus DM3PEN.

**Figure 2:**
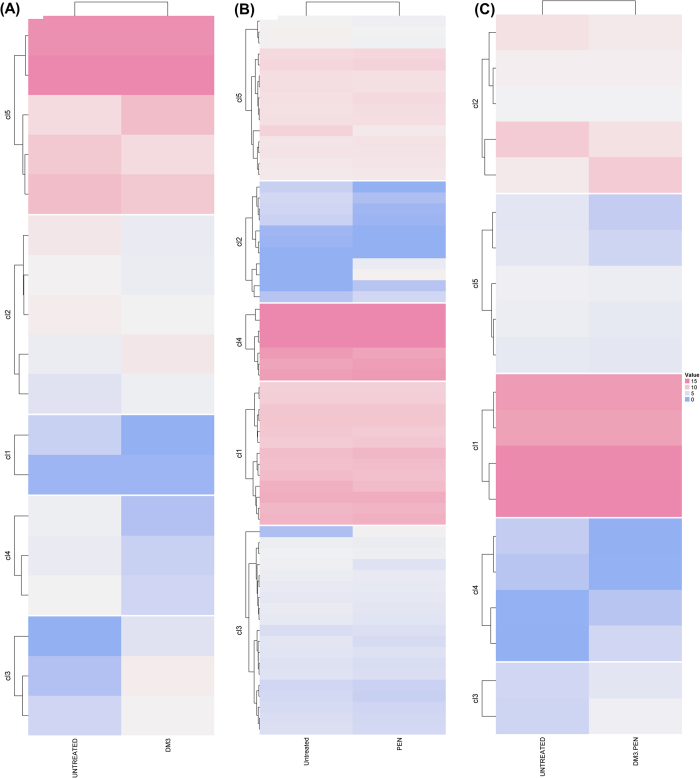
Heatmaps showing expression level of clustered genes of PSSP. Each group is classified into five clusters. (**A**) untreated versus DM3, (**B**) untreated versus PEN, and (**C**) untreated versus DM3PEN.

In this study, both PEN-resistant *S. pneumoniae* (PRSP) and PEN-susceptible *S. pneumoniae* (PSSP) were treated with DM3, PEN, and DM3PEN (combination treatment) to determine the underlying differential expression of genes and associated pathways following the drug treatment. This allows us to better understand the mechanism of actions of DM3 and the synergistic effect of DM3PEN. Heatmaps showing the differential gene expression for both untreated and treated cells against PRSP and PSSP are shown in Figs 1 and 2, respectively. As compared to PSSP, sharp differences in the number of differentially expressed genes and enrichment pathways was observed. For PRSP, there are a total of 682, 721, and 695 differentially expressed genes for DM3-, PEN-, and DM3PEN-treated groups, respectively. Gene annotations (as well as statistical analysis) of the enrichment pathways can be found in supplementary Tables S1–S3. In contrast, there are only a small set of differentially expressed genes 18, 65, and 20 for DM3-, PEN-, and DM3PEN-treated PSSP, respectively. Pathway enrichment was only determined for PEN-treated group (Table S4) but not for groups treated with DM3 and DM3PEN.

### Effects of DM3 and combination treatment on amino acid metabolism

Transcriptomic analysis on both PRSP and PSSP showed that DM3 and PEN have predominant effects on pneumococcal amino acids biosynthesis processes. From the gene enrichment analyses, the precursory pathways responsible for amino acids biosynthesis were noted. These include amine (GO:0009309), nitrogen compound (GO:0044271), carboxylic acid (GO:0046394), and aromatic compound (GO:0019438) biosynthesis processes. Although the differentially expressed genes encoded for a number of amino acids were reported including glycine, alanine, glutamate, and aspartate, the aromatic and branched chain family amino acids were most affected. The branched chain amino acids were valine, leucine, and isoleucine while aromatic amino acids included phenylalanine, tyrosine, and tryptophan. Tryptophan represented the most affected amino acids among the aromatic group as the expression of high number of genes associated with tryptophan precursor anthranilate biosynthesis and metabolisms were altered. Moreover, the specific downregulation of tryptophan biosynthesis (GO:0000162) and tryptophan metabolic process (GO:6568) were due to PEN as seen in both PEN- and DM3PEN-treated groups. For alanine biosynthesis, one unique gene (SP_1671, D-alanyl-alanine synthetase A) was downregulated in both DM3 and DM3PEN-treated PRSP but not in PEN-treated group (Tables S1–S3).

PEN-treated cells observed greater pathway differences as seen with the doubling of the number of enriched pathways found as compared to the DM3-treated cells (Tables S1 and S2). Several of these were associated with indolalklyamine, indole, and indole derivatives-related pathways, heterocycle biosynthesis, chorismate metabolic process, lyase, dicarboxylic acid metabolic and biosynthetic processes. Similar results were observed in DM3PEN and this was likely be due to presence of PEN in the combination treatment which produced such effects in the cells.

For PSSP, the set of differentially expressed genes in all three groups were similar, observing predominant effect against the 30S small ribosomal subunit involving significant upregulation of the genes *rrnaB16S*, *rrnaC16S, rrnaC23S,* and *rrnaD23S*. Upregulation of *rrnaC16S* and 23S *rrnaD23S* rRNA genes were particularly drastic with more than 32-fold change as compared to the untreated cells except the lower upregulation fold-change in rrnaB16S of DM3PEN group.

### Effects of DM3 and combination treatment on nucleic acid metabolism

Results showed significant differential expression associated with genes related to DNA replication and transcription mechanisms. Notably, genes encoded for DNA helicase, gyrase, and topoisomerases subunits were differentially expressed. Different subunits of the DNA-directed RNA polymerase were found to be differentially expressed with PEN-treatment; while both alpha- and beta-subunits were upregulated, the delta-subunit was downregulated. This is accompanied by upregulation of RNA polymerase sigma factor RpoD. Conversely, RpoD was downregulated in DM3-treated cells and no differential expression was observed with DNA-binding RNA polymerase subunits indicating that DM3 has no inhibitory activity on RNA polymerase. In the combination treatment, the collective effects were noted with upregulation of DNA-directed RNA-polymerase beta subunit while both alpha- and delta were downregulated accompanied by upregulation of RpoD. Besides, all three translation-initiation factor-1 (IF-1), IF-2, and IF-3 were differentially expressed but only IF-3 was reported in DM3 treatment.

Downregulation of the alpha- and beta subunits in DNA topoisomerase IV was found in both DM3- and PEN-treatment, however, the expression of topoisomerase I was increased in DM3 but decreased in PEN-treated cells. Unlike PEN which caused increased expression in DNA gyrase, DM3 exerted no effect on this enzyme. Such differential expressions were observed in combination treatment whereby topoisomerase I was downregulated. In addition, gene enrichment performed showed transposase activity with differential expression of the IS4-like protein.

A number of unique enrichment pathways associated with nucleic acids (purine and pyrimidine) biosynthesis and metabolisms were noted with combination treatment. These were not found in the standalone DM3 and PEN treatments against pneumococci. The pathways reported in PEN were of purine nucleotide binding. Conversely, many pathways associated with nucleoside/ribonucleoside triphosphate biosynthetic/metabolic processes were observed. Examples include purine nucleoside triphosphate metabolic/biosynthetic process (GO:0009144/5), purine ribonucleoside triphosphate metabolic/biosynthetic process (GO:0009205/6), purine nucleotide metabolic/biosynthetic process (GO:0009150/2), ribonucleotide metabolic/biosynthetic process (GO:0009259/60), and others.

In addition, the downstream processes following amino acids biosynthesis leading to the generation of peptides/proteins were affected by the treatments as well. Differential RNA expressions associated with aminoacyl-tRNA biosynthesis, tRNA ligase activity, 30S and 50S ribosomal proteins, and ribosomal large subunit assembly. The translation-initiation factors (IFs) were differentially expressed in the treatment groups where (1) in DM3 treatment group, only IF3 was differentially expressed with upregulation, (2) PEN treatment group noted upregulation of IF-1 and IF-2, while IF-3 was downregulated and (3) DM3PEN was observed with IF-2 upregulation and IF-3 downregulation.

### Effects of DM3 and combination treatment on pneumococcal cell wall, pathogenesis, and competence induction

Gene enrichment analyses highlighted that genes encoding for cell membrane and transmembrane pathways were clearly impacted in DM3-treated pneumococci. More than 20 genes were differentially expressed in these pathways and represented the largest gene sets as compared to any other pathways. Such effects were similarly observed in DM3PEN group but not in PEN treatment alone. Moreover, DM3PEN-treated group was reported with changes in a number of transmembrane transport associated pathways and these include the cation transmembrane transport (GO:0034220), monovalent inorganic cation transmembrane transporter activity (GO:0015077), hydrogen ion transmembrane transporter activity (GO:0015078), and others.

In DM3-treated pneumococci, a total of eight genes were differentially expressed which included the response regulator CiaR, sensor histidine kinase CiaH, and six competence-induced proteins Ccs16, CelA, CelB, CglA, ComF, Ccs4. Among these genes, Ccs16, ComF, Ccs4, CiaR, and CiaH were downregulated. For PEN-treated group, only five differentially expressed genes (CelB, CglA, Ccs4, CiaR, CiaH) were noted at which all were downregulated. Only three genes (CelB, CglA, Ccs4, with an addition of one unique entry CoiA) were differentially expressed in the DM3PEN-treatment group. CoiA was upregulated in the combination treatment. Cells treated with DM3 alone could have greater alteration in competence regulatory activity than PEN or the combination treatment.

*S. pneumoniae* has capsular polysaccharide (CPS) covering the outer surface of the cell wall. Unlike PEN which caused downregulation in three genes CPS4A, CPS4C, CPS4D and upregulation in CPS4B, all four genes were downregulated in DM3-treated group. This CPS4B downregulatory activity was not seen in the combination treatment and is specific to the standalone DM3 treatment. Hence, DM3 could exert specific inhibitory activity against CPS4B. Suppression of both hemolysin and exfoliative toxin in *S. pneumoniae* were seen in both standalone DM3 and PEN groups, however, combination of both drugs lead to upregulation of hemolysin in the pneumococcal cells.

DM3 has no significant effect on the major protein pneumococcal autolysin but upregulation was observed in combination treatment despite being downregulated in PEN-treated group. Notably, only standalone DM3 treatment resulted in downregulation of the serine protease (SP-2239) linked to the cationic AMP resistance pathway (CAMP). This is rather unusual as conventional antibiotics would eventually select, induce, and eventually lead to expansion of the antibiotic-resistance clones of bacterial cells. Interestingly, DM3 appeared to reduce pneumococcal CAMP resistance by decreasing the expression of SP-2239, a gene responsible for cationic antimicrobial peptide resistance in pneumococcal cells.

## Discussion

Novel AMPs drug discovery have received much attentions in recent years with increasing number of engineered AMPs variants documented with potent and broad spectrum antimicrobial activity. These short peptides could be the future alternative or supportive treatment to conventional antibiotics where usage have been heavily complicated by reports of multidrug-resistance and high-level resistance microbial strains. Our previous work had designed DM3 which exhibited strong *in vitro* antipneumococcal activity against *S. pneumoniae* including the PRSP strain^37^. Subsequent *in vivo* murine infection model testing showed promising therapeutic efficacy particularly using combination treatment^38^. To further investigate the mechanism of actions of DM3, we perform high-throughput Next-generation sequencing platform using RNA-seq to study the transcriptomic profile of DM3 treatment. Differential expression profiles and gene enrichment analyses allow the statistically significant affected pathways and genes to be compared and shortlisted to investigate the treatment effects.

Pneumococcal virulence factors include a set of cell wall- or surface anchor proteins to achieve efficient colonization, invasion, and establishment. One of these is autolysin , a N-acetylmuramoyl L-alanine amidase that cleaves lactyl-amide bond linking the peptide-glycan components of peptidoglycan causing cell wall hydrolysis of the producer host. Autolysin has been described in PEN-induced lysis^39^,^40^. Increased expression of autolysin in combination treatment could have induced the autolytic mechanism in pneumococci leading to cell death. This is opposed to PEN treatment where autolysin expression was downregulated and thus suggests a different cell lysis mechanism. Our previous result based on transmission electron micrograph reported extensive cell wall and cell membrane lysis processes in DM3-treated pneumococcal cells. Together with the absence of differential autolysin expression in DM3-treated group in this study, it is suggested that other lytic mechanisms could be involved. In addition, membrane and cell wall associated structural components and transport mechanisms were greatly affected particularly in combination treatment. One example is downregulation of the transmembrane water channel protein aquaporin in both DM3 and PEN treatments, however, combination of both drugs produced synergism which observed opposing effects and caused upregulation in aquaporin gene expression.

Pneumococcal CPS differs in the chemical compositions thus giving rise to the serogroups/serotypes antigen-antisera based classification. CPS is a virulence factor that covers the outermost layer of pneumococcal cell wall serving multiple functions particularly in protection against host immune responses following invasion and protects against transport of harmful molecules into pneumococcal cells. In this study, all four subunits of CPS4ABCD were downregulated in DM3-treated cells while only CPS4ACD, but not CPS4B were downregulated in PEN and combination treatments. Standalone DM3 may have profound effects in suppressing all CPS4ABCD subunits and thus the inhibition could be of higher efficiency as compared to PEN- and DM3PEN treatments. Reduction in CPS gene expression may impair pneumococcal cell’s host immune evasion resulting in higher susceptibility to phagocytosis and greater clearance efficiency^41^. Besides, DM3 showed predominant cell wall and cell membrane regulatory effects and could partly contribute to explain its lytic activity in pneumococcal cells.

CAMP resistance mechanism downregulation induced by DM3 is of interest. It is hypothesized that DM3 could reduce the CAMP resistance mechanism to enhance its antimicrobial activity on the target cells. Additionally, downregulation of CAMP resistance mechanism suggests that the AMP-defense mechanism in pneumococci could have been compromised leading to increased susceptibility of *S. pneumoniae* against other AMPs classes. However, further investigations are needed to support the hypothesis.

DM3 treatment was found to alter competence regulatory activity in *S. pneumoniae*. The number of differentially expressed genes in DM3 treated cells were higher than PEN treated cells. Natural competence induction in *S. pneumoniae* is a quorum-sensing regulated transient mechanism encoded by *comCDE* and *comAB* regulons in allowing the cells to undergo genetic transformation by uptake of foreign DNA^42^. *comCDE* is responsible for induction of genetic competence *comC* encodes for the pheromone-like competence-stimulating peptide (CSP) which is exported using the ComAB transporter^43^. The CSP stimulation signal will be captured by the ComD/ComE signal transduction system^44^. Another gene, *endA*, is also a membrane-bound DNA-entry nuclease important in pneumococcal transformation. However, DM3 as well as the combination treatment DM3PEN showed no significant effect on these fundamental competence genes responsible for competent induction. Significant changes in expression are reported with *celA*, *celB*, *cglA*, *ccs4* and others exhibiting important roles in DNA transport, processing, and recombination; CelA and Cel B are both DNA transformation transporter CglA while *ccs4* and *ccs16* are competence-induced proteins. Competence regulation was not the primary target of DM3 in standalone or in combination with PEN. Of note, CoiA, which functions to promote genetic recombination during transformation^45^, was only found in cells treated with DM3PEN suggesting the unique effect of the synergistic treatment in enhancing transformational recombination. Additionally, it is possible that the current experimental design could have undermined the effect of DM3 on competence induction and represents one of the limitation of the current study. This is because the pneumococcal culture used was in mid-log phase rather than at the beginning of log phase to which pneumococcus is at the highest competence capacity^46^. Although current study was not directed at investigating the role of DM3 in regulation of transformation in *S. pneumoniae*, this could be an aspect to study about DM3 in the future.

D-alanine metabolism was only found in cells treated with DM3 whether in standalone or combination, hence DM3 is hypothesized to exert inhibitory effect on the processing of D-alanine which is an important intermediate in cell wall biosynthesis. The lower expression of this component could result in cell wall lysis and cell death.

There are many heavily affected genes and pathways which are common to all three treatments. These include specific pathways collected under purine and pyrimidine biosynthesis and metabolisms, aminoacyl-tRNA biosynthesis, rRNA, ribosomal proteins, ATP biosynthesis and metabolisms, ABC transport system, and the phophotransferase (PTS) system. One potential explanation is these pathways constitute the common sets of genes and pathways in response to antimicrobial treatment. Replication, transcription, and translation mechanisms have seen a number of changes arising from the treatments as well. For example, RpoD was downregulated in the DM3-treated cells but otherwise no effect on RNA polymerase. On the contrary, combination treatment using both DM3 and PEN caused downregulation of RNA polymerase accompanied by RpoD upregulation which was in part due to the combination effect from PEN-treatment. While PEN increased DNA gyrase expression in pneumococcal cells, this was not the target of DM3. Notably, DM3PEN caused downregulation of topoisomerase I which could affect the mechanism of DNA replication and transcription.

From the study, several important genes and its associated pathways affected by DM3 and DM3PEN have been highlighted. This provides a better understanding of the drug effects at the genomic level. Together with our previous study, it is becoming clearer that DM3 exerts multiple inhibitory mechanisms by direct cell wall or cell membrane lysis killing of the target bacterial cells enhanced with disruption mechanisms to inhibit cell wall biosynthetic processes. In addition, DM3 antibacterial activity is supported by metabolic disruption activities to produce higher antibacterial efficiency. The two main metabolic processes affected are nucleic acid and amino acid biosynthesis activities. Thus, DM3 is a potent antibacterial targeting multiple cellular targets to exert killing effects. Moreover, it is important to state that the greater extent of differentially expressed genes and pathways involved due to DM3PEN-treatment may be the main reason why DM3-PEN combination showed better therapeutic efficiency in an *in vivo* infection model^38^.

One interesting point to highlight is the higher susceptibility of PRSP than PSSP to DM3. This is essentially one of our main objectives in designing DM3 – a novel drug possessing high antibacterial activity against the antibiotic-resistance strains. The gene expression profile of SP17 (PRSP strain used in this study) was heavily affected by DM3 and DM3PEN which was in sharp contrast to the gene expression profile of SP27 (PSSP strain used in this study). This strongly suggests that DM3 could have higher inhibitory effect against PRSP than PSSP but it is unclear of why such differences exists whether it is due to PEN-susceptibility of the strains alone or involve other factors including serotype variation, thickness of cell wall, pathogenicity of strain, and others. We are unclear of how these complex interactions (up or downregulation) occur in the pneumococcal cells at this stage. Therefore, further studies to determine the key mechanisms causing cell death based on *in vitro* experiments is proposed in subsequent investigations.

## Methods

### Peptide Synthesis

DM3 was synthesized by Genscript Inc. (USA) using Fluorenylmethyloxycarbonyl chloride (Fmoc) chemistry to >90% purity and validated using High Performance Liquid Chromatography and Mass Spectrometry.

### Pneumococcal cultures and assay media

One PSSP (SP17, MIC_PEN_ = 0.06 μg/ml) and –resistant (SP27, MIC_PEN_ = 4 μg/ml) isolates were selected from the previous collection maintained in the laboratory. The isolates were stored in multiple vials in BHI supplemented with 10% glycerol at −80 °C to avoid repeated freeze-thaw cycles. The isolates were passaged twice prior to experimentation. All experimental were carried out in accordance with approved guidelines and were approved by the University Malaya Biosafety & Biosecurity Committee.

### Cell treatment and RNA extraction

Overnight bacterial cultures on defibrinated sheep blood agar (Oxoid, UK) were inoculated into Mueller-Hinton Broth (MHB) broth using direct colony suspension method and incubated at 37 °C with 5% CO_2_ and shaked at 200 rpm for 4–6 hrs to mid-log phase growth (approx. OD_600_ 0.35–0.5). Aliquot equivalent to 2 × 10^9^ CFU was transferred into a fresh tube and make up to 10 ml with fresh MHB. Both PRSP (SP17, serotype 19F) and PSSP (SP27, serogroup 18) strains used were treated at the respective MIC levels for 60 min: SP17, DM3 (31.25 μg/ml), PEN (4 μg/ml), and DM3 + PEN (7.81 μg/ml, 0.5 μg/ml); SP27, DM3 (31.25 μg/ml), PEN (0.06 μg/ml), and DM3 + PEN (7.81 μg/ml, 0.015 μg/ml). The 60 min treatment duration was chosen as it was found that prolonged treatment for 120 min or more caused low yield and poor quality of RNA obtained probably due to direct lysis of cells by DM3 and hence release of cellular contents including RNA to the medium before RNA extraction. The short treatment duration would still allow substantial interruption of expression changes in the cells. Untreated cells was served as control. Subsequently, the suspensions were washed twice and resuspended in one volume of PBS followed by addition of two volumes of RNAprotect Bacteria reagent (Qiagen, Germany), immediately vortex mixed for 5 s and incubated at room temperature for 5 min before centrifuge pelleting at 5000× g for 10 min and discarded the supernatant. The pellets were lysed with 20 μl Proteinase K (Qiagen, Germany) and 200 μl bacterial lysis mix consisting of mutanolysin (M9901, Sigma, US) and lysozyme to final concentrations of 15 mg/ml and 15 U/ml, respectively made up to 200 μl using Tris-EDTA buffer. The suspensions were vortexed for 10 s and incubated at room temperature for 10 min with 2 min mixing interval. RNA extraction of the treated cells was performed using RNeasy Plus Mini kit (Qiagen, Germany) according to manufacturer’s guidelines and eluted in DEPC water (Bioline, UK). A total of three biological replicates were included for each treatment group and an untreated control group for comparison analysis.

### RNA-Seq library preparation and analysis

Quality of RNA was verified using Bioanalyzer (Agilent Technologies, Santa Clara, CA, USA) and NanoDrop spectrometer. Samples that passes quality control (minimum RNA integrity number (RIN) of 7, absorbance ratios A260/280 in the range 2.0–2.2 and A260/230 above 1.8), a non-normalized cDNA library was constructed. Barcoded libraries were multiplexed by 12 in each lane and sequenced on an Illumina HiSeq 2000 system using the single-end mode. The length of the reads was around 100 bp. Quality control of the RNA-Seq data was performed using FastQC and detailed information about the quality of reads in each replicate is provided in Additional file (xx_). Sequence reads have been deposited in the NCBI Sequence Read Archive (SRA) under accession number PRJNA308880 (www.ncbi.nlm.gov/bioproject/PRJNA308880).

### Quality check (QC) with FastQC

Adapters from the fastQ file were removed using Cutadapt (https://code.google.com/p/cutadapt/). Removal of reads with phred score below 20 were performed using fastx-toolkit (http://hannonlab.cshl.edu/fastx_toolkit/).

### Mapping and Expression analysis

Raw reads in fastq format from illumina sequencing were used to map against *streptococcus pneumoniae* TIGR4 genome (NC_003028) by TopHat v2.0.10 program^47^. To compare expression analysis among samples output bam file from TopHat and GFF file from gene prediction were used as input to cuffdiff v2.1.1 program^48^ with classic method of normalization with FPKM to identify the differentially expressed genes between all the samples.

### Gene clustering and Heat map

Differentially expressed genes were clustered using K-means clustering algorithm using ComplexHeatmap^41^ package from Bioconductor in R. Clusters generated by K-means were submitted to DAVID 6.7 web server^40^ for gene enrichment studies. Annotations from various databases such as KEGG pathways, gene ontology (GO), and swissprot were also retrieved from DAVID 6.7 server^40^.

## Additional Information

**How to cite this article**: Le, C.-F. *et al.* Transcriptome analysis of *Streptococcus pneumoniae* treated with the designed antimicrobial peptides, DM3. *Sci. Rep.* **6**, 26828; doi: 10.1038/srep26828 (2016).

## Supplementary Material

Supplementary Information

Supplementary Information

Supplementary Information

Supplementary Information
