# Supplementary material for: Transcriptome analysis of Streptococcus pneumoniae treated with the designed antimicrobial peptides, DM3
Source: Sci Rep. 2016 May 26;6:26828. doi: 10.1038/srep26828 (PMC4881017; doi:10.1038/srep26828)
Supplement: Supplementary Information [file srep26828-s1.pdf]

Transcriptome analysis of *Streptococcus pneumoniae* treated with the designed antimicrobial peptides, DM3

Cheng-Foh Le, Ranganath Gudimella, Rozaimi Razali, Rishya Manikam & Shamala Devi Sekaran

Table S1. Pathway enrichment for PRSP comparing between with and without DM3 treatment.

Annotation Cluster 1	Enrichment Score: 2.6201033536120835	
Category	Term	Genes
KEGG_PATHWAY	spn00290:Valine, leucine and isoleucine biosynthesis	SP_1255, SP_1258, SP_0450, SP_0445
GOTERM_BP_FAT	GO:0008652~cellular amino acid biosynthetic process	SP_1296, SP_1255, SP_1013, SP_1258, SP_1813, SP_1812, SP_0450, SP_0289, SP_0445
GOTERM_BP_FAT	GO:0009309~amine biosynthetic process	SP_1296, SP_1255, SP_1013, SP_1258, SP_1813, SP_1812, SP_0450, SP_0289, SP_0445
GOTERM_BP_FAT	GO:0046394~carboxylic acid biosynthetic process	SP_1296, SP_1255, SP_1013, SP_1258, SP_1813, SP_1812, SP_0450, SP_0289, SP_0445
GOTERM_BP_FAT	GO:0016053~organic acid biosynthetic process	SP_1296, SP_1255, SP_1013, SP_1258, SP_1813, SP_1812, SP_0450, SP_0289, SP_0445
GOTERM_BP_FAT	GO:0044271~nitrogen compound biosynthetic process	SP_0963, SP_1296, SP_0729, SP_1255, SP_1470, SP_1013, SP_1258, SP_1813, SP_1812, SP_0450, SP_0289, SP_0445
GOTERM_BP_FAT	GO:0009081~branched chain family amino acid metabolic process	SP_1255, SP_1258, SP_0450, SP_0445
GOTERM_BP_FAT	GO:0009082~branched chain family amino acid biosynthetic process	SP_1255, SP_1258, SP_0450, SP_0445
GOTERM_BP_FAT	GO:0019438~aromatic compound biosynthetic process	SP_1296, SP_1470, SP_1813, SP_1812, SP_0289
SP_PIR_KEYWORDS	amino-acid biosynthesis	SP_1255, SP_1813, SP_1812, SP_0445
GOTERM_MF_FAT	GO:0048037~cofactor binding	SP_0963, SP_1013, SP_1393, SP_1812, SP_0450, SP_0445

Annotation Cluster 2	Enrichment Score: 0.7476633689731316	
Category	Term	Genes
INTERPRO	IPR002559:Transposase, IS4-like	SP_1195, SP_1729, SP_0538
GOTERM_BP_FAT	GO:0006313~transposition, DNA-mediated	SP_1195, SP_1729, SP_0538
GOTERM_BP_FAT	GO:0032196~transposition	SP_1195, SP_1729, SP_0538
GOTERM_MF_FAT	GO:0004803~transposase activity	SP_1195, SP_1729, SP_0538
GOTERM_BP_FAT	GO:0006259~DNA metabolic process	SP_0954, SP_1195, SP_1908, SP_1729, SP_0538
GOTERM_BP_FAT	GO:0006310~DNA recombination	SP_1195, SP_1729, SP_0538
GOTERM_MF_FAT	GO:0003677~DNA binding	SP_0954, SP_1195, SP_1908, SP_1729, SP_0538

Annotation Cluster 3	Enrichment Score: 1.9779407749292082	
Category	Term	Genes
SP_PIR_KEYWORDS	membrane	a
SP_PIR_KEYWORDS	cell membrane	SP_0152, SP_0151, SP_0823, SP_0091, SP_2184, SP_1502, SP_1975, SP_0601, SP_1010, SP_2109, SP_2185, SP_0090, SP_0366, SP_1652, SP_1294, SP_2022, SP_1869, SP_1527, SP_1896, SP_1597, SP_1870, SP_1895, SP_0855
SP_PIR_KEYWORDS	transmembrane	SP_0152, SP_0823, SP_1154, SP_0091, SP_2184, SP_1502, SP_1975, SP_0601, SP_1010, SP_2109, SP_0090, SP_0664, SP_1652, SP_1294, SP_2022, SP_1869, SP_1896, SP_1597, SP_1870, SP_1895
GOTERM_CC_FAT	GO:0005886~plasma membrane	SP_0152, SP_0151, SP_0823, SP_0091, SP_2184, SP_1502, SP_1975, SP_0601, SP_1010, SP_2109, SP_2185, SP_0090, SP_0366, SP_1652, SP_1294, SP_2022, SP_1869, SP_1527, SP_1896, SP_1597, SP_1870, SP_1895, SP_0855
SP_PIR_KEYWORDS	transport	SP_0152, SP_0151, SP_0823, SP_0091, SP_2184, SP_1502, SP_1010, SP_2109, SP_0090, SP_0366, SP_2022, SP_1869, SP_1870, SP_1896, SP_1527, SP_1895
INTERPRO	IPR000515:Binding-protein-dependent transport systems inner membrane component	SP_0152, SP_0823, SP_0091, SP_1502, SP_1896, SP_2109, SP_0090, SP_1895
GOTERM_CC_FAT	GO:0031224~intrinsic to membrane	SP_0152, SP_1154, SP_0091, SP_1010, SP_0090, SP_2075, SP_1527, SP_1870, SP_1896, SP_1895, SP_0823, SP_1067, SP_2184, SP_1502, SP_0325, SP_1975, SP_0601, SP_2109, SP_0664, SP_0366, SP_1652,

		SP_1294, SP_2022, SP_1869, SP_1597, SP_1840
GOTERM_CC_FAT	GO:0016021~integral to membrane	SP_0152, SP_0823, SP_1067, SP_1154, SP_0091, SP_2184, SP_1502, SP_0325, SP_1975, SP_0601, SP_1010, SP_2109, SP_0664, SP_0090, SP_1652, SP_1294, SP_2022, SP_2075, SP_1869, SP_1896, SP_1597, SP_1870, SP_1840, SP_1895
Annotation Cluster 4	Enrichment Score: 1.7386749661077125	
Category	Term	Genes
SP_PIR_KEYWORDS	metal-binding	SP_2228, SP_1283, SP_1008, SP_0724, SP_0762, SP_0717, SP_0176, SP_1650, SP_0519, SP_2030, SP_0829
GOTERM_MF_FAT	GO:0043167~ion binding	SP_2228, SP_1283, SP_0060, SP_0178, SP_1008, SP_0797, SP_0762, SP_0717, SP_0648, SP_0176, SP_2107, SP_1650, SP_2068, SP_2030, SP_2205, SP_1297, SP_2026, SP_1894, SP_0519, SP_0962, SP_0829
GOTERM_MF_FAT	GO:0043169~cation binding	SP_2228, SP_1283, SP_0060, SP_0178, SP_1008, SP_0797, SP_0762, SP_0717, SP_0648, SP_0176, SP_2107, SP_1650, SP_2068, SP_2030, SP_2205, SP_1297, SP_2026, SP_1894, SP_0519, SP_0962, SP_0829
GOTERM_MF_FAT	GO:0046872~metal ion binding	SP_2228, SP_1283, SP_0178, SP_1008, SP_0797, SP_0762, SP_0717, SP_0176, SP_1650, SP_2068, SP_2030, SP_2205, SP_1297, SP_2026, SP_0519, SP_0962, SP_0829
GOTERM_MF_FAT	GO:0046914~transition metal ion binding	SP_2205, SP_1283, SP_0178, SP_1008, SP_1297, SP_0797, SP_0762, SP_0176, SP_1650, SP_0519, SP_2068, SP_0829
SP_PIR_KEYWORDS	zinc	SP_1283, SP_1008, SP_0176, SP_1650, SP_0519
GOTERM_MF_FAT	GO:0008270~zinc ion binding	SP_1283, SP_0178, SP_1008, SP_0797, SP_0176, SP_1650, SP_0519, SP_2068
Annotation Cluster 5	Enrichment Score: 3.674256530589223	
Category	Term	Genes
KEGG_PATHWAY	spn00061:Fatty acid biosynthesis	SP_0426, SP_0427, SP_0417, SP_0422, SP_0423
SP_PIR_KEYWORDS	Fatty acid biosynthesis	SP_0426, SP_0427, SP_0417
SP_PIR_KEYWORDS	lipid synthesis	SP_0426, SP_0427, SP_0417
SP_PIR_KEYWORDS	cytoplasm	SP_0266, SP_0426, SP_0427, SP_0417, SP_0516, SP_0798, SP_1577
