# Supplementary material for: Transcriptome analysis of Streptococcus pneumoniae treated with the designed antimicrobial peptides, DM3
Source: Sci Rep. 2016 May 26;6:26828. doi: 10.1038/srep26828 (PMC4881017; doi:10.1038/srep26828)
Supplement: Supplementary Information [file srep26828-s2.pdf]

Transcriptome analysis of *Streptococcus pneumoniae* treated with the designed antimicrobial peptides, DM3

Cheng-Foh Le, Ranganath Gudimella, Rozaimi Razali, Rishya Manikam & Shamala Devi Sekaran

Table S2. Pathway enrichment for PRSP comparing between with and without PEN treatment.

Annotation Cluster 1	Enrichment Score: 1.4416437477212627	
Category	Term	Genes
GOTERM_MF_FAT	GO:0017076~purine nucleotide binding	SP_0724, SP_1982, SP_0820, SP_0600, SP_1219, SP_1653, SP_0604, SP_0338, SP_0720, SP_1523, SP_0516, SP_0519, SP_1276, SP_1051, SP_1749, SP_1087
GOTERM_MF_FAT	GO:0030554~adenyl nucleotide binding	SP_0724, SP_1982, SP_0820, SP_0600, SP_1219, SP_1653, SP_0604, SP_0338, SP_0720, SP_1523, SP_0516, SP_0519, SP_1276, SP_1051, SP_1087
GOTERM_MF_FAT	GO:0001883~purine nucleoside binding	SP_0724, SP_1982, SP_0820, SP_0600, SP_1219, SP_1653, SP_0604, SP_0338, SP_0720, SP_1523, SP_0516, SP_0519, SP_1276, SP_1051, SP_1087
GOTERM_MF_FAT	GO:0001882~nucleoside binding	SP_0724, SP_1982, SP_0820, SP_0600, SP_1219, SP_1653, SP_0604, SP_0338, SP_0720, SP_1523, SP_0516, SP_0519, SP_1276, SP_1051, SP_1087
GOTERM_MF_FAT	GO:0032555~purine ribonucleotide binding	SP_1982, SP_0724, SP_1523, SP_0820, SP_0600, SP_1219, SP_0519, SP_0604, SP_1653, SP_1276, SP_1749, SP_0338, SP_1051, SP_1087, SP_0720
GOTERM_MF_FAT	GO:0032553~ribonucleotide binding	SP_1982, SP_0724, SP_1523, SP_0820, SP_0600, SP_1219, SP_0519, SP_0604, SP_1653, SP_1276, SP_1749, SP_0338, SP_1051, SP_1087, SP_0720
SP_PIR_KEYWORDS	atp-binding	SP_0724, SP_1523, SP_0820, SP_0600, SP_1219, SP_1653, SP_1276, SP_0604, SP_0338, SP_1087, SP_0720
GOTERM_MF_FAT	GO:0000166~nucleotide binding	SP_0724, SP_1982, SP_0820, SP_0600, SP_1219, SP_1653, SP_0604, SP_0338, SP_0720, SP_1523, SP_0516, SP_0519, SP_1276, SP_1051, SP_1749, SP_1087
SP_PIR_KEYWORDS	nucleotide-binding	SP_0724, SP_1523, SP_0820, SP_0600, SP_1219, SP_1653, SP_1276, SP_0604, SP_0338, SP_1087, SP_0720
GOTERM_MF_FAT	GO:0005524~ATP binding	SP_1982, SP_0724, SP_1523, SP_0820, SP_0600, SP_1219, SP_0519, SP_0604, SP_1653, SP_1276, SP_0338, SP_1051, SP_1087, SP_0720
GOTERM_MF_FAT	GO:0032559~adenyl ribonucleotide binding	SP_1982, SP_0724, SP_1523, SP_0820, SP_0600, SP_1219, SP_0519, SP_0604, SP_1653, SP_1276, SP_0338, SP_1051, SP_1087, SP_0720

Annotation Cluster 2	Enrichment Score: 1.867623717302853

Category	Term	Genes
KEGG_PATHWAY	spn00400:Phenylalanine, tyrosine and tryptophan biosynthesis	SP_1817, SP_1816, SP_1813, SP_1812
GOTERM_BP_FAT	GO:0000162~tryptophan biosynthetic process	SP_1817, SP_1813, SP_1812
GOTERM_BP_FAT	GO:0006568~tryptophan metabolic process	SP_1817, SP_1813, SP_1812
GOTERM_BP_FAT	GO:0006586~indolalkylamine metabolic process	SP_1817, SP_1813, SP_1812
GOTERM_BP_FAT	GO:0042434~indole derivative metabolic process	SP_1817, SP_1813, SP_1812
GOTERM_BP_FAT	GO:0042430~indole and derivative metabolic process	SP_1817, SP_1813, SP_1812
GOTERM_BP_FAT	GO:0042435~indole derivative biosynthetic process	SP_1817, SP_1813, SP_1812
GOTERM_BP_FAT	GO:0046219~indolalkylamine biosynthetic process	SP_1817, SP_1813, SP_1812
GOTERM_BP_FAT	GO:0006576~biogenic amine metabolic process	SP_1817, SP_1813, SP_1812
GOTERM_BP_FAT	GO:0042401~biogenic amine biosynthetic process	SP_1817, SP_1813, SP_1812
GOTERM_BP_FAT	GO:0042398~cellular amino acid derivative biosynthetic process	SP_1817, SP_1813, SP_1812
GOTERM_BP_FAT	GO:0006575~cellular amino acid derivative metabolic process	SP_1817, SP_1813, SP_1812
GOTERM_BP_FAT	GO:0008652~cellular amino acid biosynthetic process	SP_0825, SP_1817, SP_1813, SP_1812, SP_0450
GOTERM_BP_FAT	GO:0009309~amine biosynthetic process	SP_0825, SP_1817, SP_1813, SP_1812, SP_0450
GOTERM_BP_FAT	GO:0018130~heterocycle biosynthetic process	SP_0825, SP_1817, SP_1813, SP_1812
GOTERM_BP_FAT	GO:0009072~aromatic amino acid family metabolic process	SP_1817, SP_1813, SP_1812
GOTERM_BP_FAT	GO:0009073~aromatic amino acid family biosynthetic process	SP_1817, SP_1813, SP_1812
GOTERM_BP_FAT	GO:0046417~chorismate metabolic process	SP_1817, SP_1813, SP_1812
GOTERM_BP_FAT	GO:0046394~carboxylic acid biosynthetic	SP_0825, SP_1817, SP_1813, SP_1812, SP_0450

	process	
GOTERM_BP_FAT	GO:0016053~organic acid biosynthetic process	SP_0825, SP_1817, SP_1813, SP_1812, SP_0450
SP_PIR_KEYWORDS	amino-acid biosynthesis	SP_0825, SP_1813, SP_1812
SP_PIR_KEYWORDS	lyase	SP_1817, SP_1816, SP_1812, SP_0450
GOTERM_BP_FAT	GO:0043648~dicarboxylic acid metabolic process	SP_1817, SP_1813, SP_1812
GOTERM_BP_FAT	GO:0044271~nitrogen compound biosynthetic process	SP_0963, SP_0964, SP_0825, SP_1817, SP_1813, SP_1812, SP_0450
GOTERM_BP_FAT	GO:0019438~aromatic compound biosynthetic process	SP_1817, SP_1813, SP_1812

Annotation Cluster 3 Enrichment Score: 3.072090502555782

Category	Term	Genes
KEGG_PATHWAY	spn00061:Fatty acid biosynthesis	SP_0426, SP_0427, SP_0417, SP_0423
SP_PIR_KEYWORDS	Fatty acid biosynthesis	SP_0426, SP_0427, SP_0417
SP_PIR_KEYWORDS	lipid synthesis	SP_0426, SP_0427, SP_0417
SP_PIR_KEYWORDS	cytoplasm	SP_0266, SP_0426, SP_0427, SP_1970, SP_0417, SP_0798, SP_1577

Annotation Cluster 4 Enrichment Score: 2.293770006012736

Category	Term	Genes
KEGG_PATHWAY	spn02060:Phosphotransferase system (PTS)	SP_0061, SP_0645, SP_0877, SP_0063, SP_0647, SP_0646, SP_0062, SP_0064
KEGG_PATHWAY	spn00051:Fructose and mannose metabolism	SP_0061, SP_0877, SP_0876, SP_0063, SP_0062, SP_0064
GOTERM_BP_FAT	GO:0008643~carbohydrate transport	SP_0061, SP_0645, SP_0877, SP_0063, SP_0647, SP_0646, SP_0062, SP_2108, SP_2109, SP_0348, SP_0064
GOTERM_BP_FAT	GO:0009401~phosphoenolpyruvate-	SP_0061, SP_0645, SP_0877, SP_0063, SP_0647, SP_0646, SP_0062, SP_0064

	dependent sugar phosphotransferase system	

Annotation Cluster 5 Enrichment Score: 2.3391055181404625

Category	Term	Genes
GOTERM_BP_FAT	GO:0008652~cellular amino acid biosynthetic process	SP_0447, SP_1296, SP_2077, SP_1013, SP_1258, SP_0856, SP_0411, SP_0289, SP_0933, SP_2066, SP_0445
KEGG_PATHWAY	spn00770:Pantothenate and CoA biosynthesis	SP_0447, SP_1230, SP_0856, SP_0445
GOTERM_BP_FAT	GO:0009309~amine biosynthetic process	SP_0447, SP_1296, SP_2077, SP_1013, SP_1258, SP_0856, SP_0411, SP_0289, SP_0933, SP_2066, SP_0445
KEGG_PATHWAY	spn00290:Valine, leucine and isoleucine biosynthesis	SP_0447, SP_1258, SP_0856, SP_0445
GOTERM_BP_FAT	GO:0046394~carboxylic acid biosynthetic process	SP_0447, SP_1296, SP_2077, SP_1013, SP_1258, SP_0856, SP_0411, SP_0289, SP_0933, SP_2066, SP_0445
GOTERM_BP_FAT	GO:0016053~organic acid biosynthetic process	SP_0447, SP_1296, SP_2077, SP_1013, SP_1258, SP_0856, SP_0411, SP_0289, SP_0933, SP_2066, SP_0445
GOTERM_BP_FAT	GO:0009082~branched chain family amino acid biosynthetic process	SP_0447, SP_1258, SP_0856, SP_0445
GOTERM_BP_FAT	GO:0009081~branched chain family amino acid metabolic process	SP_0447, SP_1258, SP_0856, SP_0445
SP_PIR_KEYWORDS	branched-chain amino acid biosynthesis	SP_0447, SP_0856, SP_0445
GOTERM_BP_FAT	GO:0044271~nitrogen compound biosynthetic process	SP_0447, SP_1208, SP_2077, SP_1013, SP_1470, SP_0445, SP_2066, SP_0933, SP_0289, SP_1296, SP_0729, SP_1258, SP_0856, SP_0411
SP_PIR_KEYWORDS	amino-acid biosynthesis	SP_0447, SP_2077, SP_0856, SP_0445
