# Supplementary material for: Transcriptome analysis of Streptococcus pneumoniae treated with the designed antimicrobial peptides, DM3
Source: Sci Rep. 2016 May 26;6:26828. doi: 10.1038/srep26828 (PMC4881017; doi:10.1038/srep26828)
Supplement: Supplementary Information [file srep26828-s3.pdf]

Transcriptome analysis of *Streptococcus pneumoniae* treated with the designed antimicrobial peptides, DM3

Cheng-Foh Le, Ranganath Gudimella, Rozaimi Razali, Rishya Manikam & Shamala Devi Sekaran

Table S3. Pathway enrichment for PRSP comparing between with and without DM3PEN treatment.

Annotation Cluster 1	Enrichment Score: 2.4729993566624273	
Category	Term	Genes
KEGG_PATHWAY	spn00770:Pantothenate and CoA biosynthesis	SP_0447, SP_1231, SP_1230, SP_0856, SP_0445
KEGG_PATHWAY	spn00290:Valine, leucine and isoleucine biosynthesis	SP_0447, SP_1258, SP_0856, SP_0445
GOTERM_BP_FAT	GO:0008652~cellular amino acid biosynthetic process	SP_0447, SP_1296, SP_1013, SP_1258, SP_0931, SP_0856, SP_0289, SP_0933, SP_2066, SP_0445
GOTERM_BP_FAT	GO:0009309~amine biosynthetic process	SP_0447, SP_1296, SP_1013, SP_1258, SP_0931, SP_0856, SP_0289, SP_0933, SP_2066, SP_0445
GOTERM_BP_FAT	GO:0046394~carboxylic acid biosynthetic process	SP_0447, SP_1296, SP_1013, SP_1258, SP_0931, SP_0856, SP_0289, SP_0933, SP_2066, SP_0445
GOTERM_BP_FAT	GO:0016053~organic acid biosynthetic process	SP_0447, SP_1296, SP_1013, SP_1258, SP_0931, SP_0856, SP_0289, SP_0933, SP_2066, SP_0445
GOTERM_BP_FAT	GO:0009082~branched chain family amino acid biosynthetic process	SP_0447, SP_1258, SP_0856, SP_0445
GOTERM_BP_FAT	GO:0009081~branched chain family amino acid metabolic process	SP_0447, SP_1258, SP_0856, SP_0445
SP_PIR_KEYWORDS	branched-chain amino acid biosynthesis	SP_0447, SP_0856, SP_0445
GOTERM_BP_FAT	GO:0044271~nitrogen compound biosynthetic process	SP_0447, SP_1296, SP_1208, SP_0729, SP_1470, SP_1013, SP_1258, SP_0931, SP_0856, SP_0289, SP_0933, SP_2066, SP_0445
SP_PIR_KEYWORDS	amino-acid biosynthesis	SP_0447, SP_0931, SP_0856, SP_0445
Annotation Cluster 2	Enrichment Score: 3.4026998493233367	
Category	Term	Genes
KEGG_PATHWAY	spn00520:Amino sugar and nucleotide sugar	SP_0061, SP_1607, SP_0063, SP_0062, SP_1685, SP_1852,

	metabolism	SP_1675, SP_1853, SP_1676, SP_0284, SP_0064
KEGG_PATHWAY	spn02060:Phosphotransferase system (PTS)	SP_0061, SP_0645, SP_0063, SP_0647, SP_0646, SP_0062, SP_0284, SP_0064
KEGG_PATHWAY	spn00051:Fructose and mannose metabolism	SP_0061, SP_0876, SP_0063, SP_0062, SP_0284, SP_0064
GOTERM_BP_FAT	GO:0008643~carbohydrate transport	SP_0061, SP_0645, SP_0063, SP_0647, SP_0646, SP_0062, SP_2108, SP_2109, SP_0284, SP_0348, SP_0064
GOTERM_BP_FAT	GO:0009401~phosphoenolpyruvate-dependent sugar phosphotransferase system	SP_0061, SP_0645, SP_0063, SP_0647, SP_0646, SP_0062, SP_0284, SP_0064

Annotation Cluster 3	Enrichment Score: 2.110469349865255	
Category	Term	Genes
KEGG_PATHWAY	spn00400:Phenylalanine, tyrosine and tryptophan biosynthesis	SP_1817, SP_1816, SP_1813, SP_1812
GOTERM_BP_FAT	GO:0000162~tryptophan biosynthetic process	SP_1817, SP_1813, SP_1812
GOTERM_BP_FAT	GO:0006568~tryptophan metabolic process	SP_1817, SP_1813, SP_1812
GOTERM_BP_FAT	GO:0006586~indolalkylamine metabolic process	SP_1817, SP_1813, SP_1812
GOTERM_BP_FAT	GO:0042434~indole derivative metabolic process	SP_1817, SP_1813, SP_1812
GOTERM_BP_FAT	GO:0042430~indole and derivative metabolic process	SP_1817, SP_1813, SP_1812
GOTERM_BP_FAT	GO:0042435~indole derivative biosynthetic process	SP_1817, SP_1813, SP_1812
GOTERM_BP_FAT	GO:0046219~indolalkylamine biosynthetic process	SP_1817, SP_1813, SP_1812
GOTERM_BP_FAT	GO:0006576~biogenic amine metabolic process	SP_1817, SP_1813, SP_1812
GOTERM_BP_FAT	GO:0042401~biogenic amine biosynthetic process	SP_1817, SP_1813, SP_1812
GOTERM_BP_FAT	GO:0042398~cellular amino acid derivative biosynthetic process	SP_1817, SP_1813, SP_1812
GOTERM_BP_FAT	GO:0006575~cellular amino acid derivative metabolic process	SP_1817, SP_1813, SP_1812
GOTERM_BP_FAT	GO:0008652~cellular amino acid biosynthetic process	SP_0825, SP_1817, SP_1813, SP_1812, SP_0450
GOTERM_BP_FAT	GO:0009309~amine biosynthetic process	SP_0825, SP_1817, SP_1813, SP_1812, SP_0450
GOTERM_BP_FAT	GO:0018130~heterocycle biosynthetic process	SP_0825, SP_1817, SP_1813, SP_1812
GOTERM_BP_FAT	GO:0046394~carboxylic acid biosynthetic process	SP_0825, SP_1817, SP_1813, SP_1812, SP_0450
GOTERM_BP_FAT	GO:0016053~organic acid biosynthetic process	SP_0825, SP_1817, SP_1813, SP_1812, SP_0450

GOTERM_BP_FAT	GO:0009072~aromatic amino acid family metabolic process	SP_1817, SP_1813, SP_1812
GOTERM_BP_FAT	GO:0009073~aromatic amino acid family biosynthetic process	SP_1817, SP_1813, SP_1812
GOTERM_BP_FAT	GO:0046417~chorismate metabolic process	SP_1817, SP_1813, SP_1812
GOTERM_BP_FAT	GO:0044271~nitrogen compound biosynthetic process	SP_0963, SP_0964, SP_0825, SP_1817, SP_1813, SP_1812, SP_0450
SP_PIR_KEYWORDS	lyase	SP_1817, SP_1816, SP_1812, SP_0450
SP_PIR_KEYWORDS	amino-acid biosynthesis	SP_0825, SP_1813, SP_1812
GOTERM_BP_FAT	GO:0043648~dicarboxylic acid metabolic process	SP_1817, SP_1813, SP_1812
GOTERM_BP_FAT	GO:0019438~aromatic compound biosynthetic process	SP_1817, SP_1813, SP_1812

Annotation Cluster 4	Enrichment Score: 2.4509255440702415	
Category	Term	Genes
KEGG_PATHWAY	spn00190:Oxidative phosphorylation	SP_1512, SP_1510, SP_1513, SP_1514, SP_1508, SP_1509
SP_PIR_KEYWORDS	atp synthesis	SP_1512, SP_1510, SP_1513, SP_1514, SP_1508, SP_1509
SP_PIR_KEYWORDS	Hydrogen ion transport	SP_1512, SP_1510, SP_1513, SP_1514, SP_1508, SP_1509
GOTERM_CC_FAT	GO:0045259~proton-transporting ATP synthase complex	SP_1512, SP_1510, SP_1513, SP_1514, SP_1508, SP_1509
GOTERM_MF_FAT	GO:0046933~hydrogen ion transporting ATP synthase activity, rotational mechanism	SP_1512, SP_1510, SP_1513, SP_1514, SP_1508, SP_1509
SP_PIR_KEYWORDS	ion transport	SP_1512, SP_1510, SP_1513, SP_1514, SP_1508, SP_1509
GOTERM_MF_FAT	GO:0015077~monovalent inorganic cation transmembrane transporter activity	SP_1512, SP_1510, SP_1513, SP_1514, SP_1508, SP_1509
GOTERM_MF_FAT	GO:0015078~hydrogen ion transmembrane transporter activity	SP_1512, SP_1510, SP_1513, SP_1514, SP_1508, SP_1509
GOTERM_BP_FAT	GO:0006119~oxidative phosphorylation	SP_1512, SP_1510, SP_1513, SP_1514, SP_1508, SP_1509
GOTERM_BP_FAT	GO:0006818~hydrogen transport	SP_1512, SP_1510, SP_1513, SP_1514, SP_1508, SP_1509
GOTERM_BP_FAT	GO:0015992~proton transport	SP_1512, SP_1510, SP_1513, SP_1514, SP_1508, SP_1509
GOTERM_BP_FAT	GO:0015985~energy coupled proton transport, down electrochemical gradient	SP_1512, SP_1510, SP_1513, SP_1514, SP_1508, SP_1509

GOTERM_BP_FAT	GO:0015986~ATP synthesis coupled proton transport	SP_1512, SP_1510, SP_1513, SP_1514, SP_1508, SP_1509
GOTERM_CC_FAT	GO:0016469~proton-transporting two-sector ATPase complex	SP_1512, SP_1510, SP_1513, SP_1514, SP_1508, SP_1509
GOTERM_BP_FAT	GO:0034220~ion transmembrane transport	SP_1512, SP_1510, SP_1513, SP_1514, SP_1508, SP_1509
GOTERM_BP_FAT	GO:0042777~plasma membrane ATP synthesis coupled proton transport	SP_1510, SP_1513, SP_1508, SP_1509
GOTERM_BP_FAT	GO:0006754~ATP biosynthetic process	SP_1512, SP_1510, SP_1513, SP_1514, SP_1508, SP_1509
GOTERM_BP_FAT	GO:0046034~ATP metabolic process	SP_1512, SP_1510, SP_1513, SP_1514, SP_1508, SP_1509
GOTERM_BP_FAT	GO:0009144~purine nucleoside triphosphate metabolic process	SP_1512, SP_1510, SP_1513, SP_1514, SP_1508, SP_1509
GOTERM_BP_FAT	GO:0009145~purine nucleoside triphosphate biosynthetic process	SP_1512, SP_1510, SP_1513, SP_1514, SP_1508, SP_1509
GOTERM_BP_FAT	GO:0009201~ribonucleoside triphosphate biosynthetic process	SP_1512, SP_1510, SP_1513, SP_1514, SP_1508, SP_1509
GOTERM_BP_FAT	GO:0009199~ribonucleoside triphosphate metabolic process	SP_1512, SP_1510, SP_1513, SP_1514, SP_1508, SP_1509
GOTERM_BP_FAT	GO:0009206~purine ribonucleoside triphosphate biosynthetic process	SP_1512, SP_1510, SP_1513, SP_1514, SP_1508, SP_1509
GOTERM_BP_FAT	GO:0009205~purine ribonucleoside triphosphate metabolic process	SP_1512, SP_1510, SP_1513, SP_1514, SP_1508, SP_1509
GOTERM_MF_FAT	GO:0022890~inorganic cation transmembrane transporter activity	SP_1512, SP_1510, SP_1513, SP_1514, SP_1508, SP_1509
GOTERM_BP_FAT	GO:0009142~nucleoside triphosphate biosynthetic process	SP_1512, SP_1510, SP_1513, SP_1514, SP_1508, SP_1509
SP_PIR_KEYWORDS	cf(1)	SP_1510, SP_1508, SP_1509
SP_PIR_KEYWORDS	cf(0)	SP_1512, SP_1513, SP_1514
GOTERM_BP_FAT	GO:0015672~monovalent inorganic cation transport	SP_1512, SP_1510, SP_1513, SP_1514, SP_1508, SP_1509
GOTERM_BP_FAT	GO:0009141~nucleoside triphosphate metabolic process	SP_1512, SP_1510, SP_1513, SP_1514, SP_1508, SP_1509
GOTERM_BP_FAT	GO:0055085~transmembrane transport	SP_1512, SP_1510, SP_1513, SP_1514, SP_1508, SP_1509
GOTERM_BP_FAT	GO:0006091~generation of precursor metabolites and energy	SP_1512, SP_1510, SP_1513, SP_1514, SP_1508, SP_1509
GOTERM_BP_FAT	GO:0009150~purine ribonucleotide metabolic process	SP_1512, SP_1510, SP_1513, SP_1514, SP_1508, SP_1509

GOTERM_BP_FAT	GO:0009152~purine ribonucleotide biosynthetic process	SP_1512, SP_1510, SP_1513, SP_1514, SP_1508, SP_1509
GOTERM_CC_FAT	GO:0045263~proton-transporting ATP synthase complex, coupling factor F(o)	SP_1512, SP_1513, SP_1514
GOTERM_BP_FAT	GO:0006164~purine nucleotide biosynthetic process	SP_1512, SP_1510, SP_1513, SP_1514, SP_1508, SP_1509
GOTERM_BP_FAT	GO:0016310~phosphorylation	SP_1512, SP_1510, SP_1513, SP_1514, SP_0799, SP_1508, SP_1509
GOTERM_BP_FAT	GO:0006163~purine nucleotide metabolic process	SP_1512, SP_1510, SP_1513, SP_1514, SP_1508, SP_1509
GOTERM_CC_FAT	GO:0045261~proton-transporting ATP synthase complex, catalytic core F(1)	SP_1510, SP_1508, SP_1509
GOTERM_BP_FAT	GO:0009260~ribonucleotide biosynthetic process	SP_1512, SP_1510, SP_1513, SP_1514, SP_1508, SP_1509
GOTERM_BP_FAT	GO:0009259~ribonucleotide metabolic process	SP_1512, SP_1510, SP_1513, SP_1514, SP_1508, SP_1509
SP_PIR_KEYWORDS	transport	SP_0877, SP_1512, SP_1461, SP_1510, SP_1580, SP_1778, SP_1513, SP_1514, SP_1508, SP_1509
GOTERM_BP_FAT	GO:0006793~phosphorus metabolic process	SP_1512, SP_1510, SP_1513, SP_1514, SP_0799, SP_1508, SP_1509
GOTERM_BP_FAT	GO:0006796~phosphate metabolic process	SP_1512, SP_1510, SP_1513, SP_1514, SP_0799, SP_1508, SP_1509
GOTERM_BP_FAT	GO:0034654~nucleobase, nucleoside, nucleotide and nucleic acid biosynthetic process	SP_1512, SP_1510, SP_1513, SP_1514, SP_1577, SP_1508, SP_1509
GOTERM_BP_FAT	GO:0034404~nucleobase, nucleoside and nucleotide biosynthetic process	SP_1512, SP_1510, SP_1513, SP_1514, SP_1577, SP_1508, SP_1509
SP_PIR_KEYWORDS	membrane	SP_0877, SP_0013, SP_1512, SP_1461, SP_1510, SP_1513, SP_1514, SP_0799, SP_1508, SP_1509, SP_1882
SP_PIR_KEYWORDS	cell membrane	SP_0877, SP_0013, SP_1512, SP_1461, SP_1510, SP_1513, SP_1514, SP_0799, SP_1508, SP_1509
GOTERM_MF_FAT	GO:0046961~proton-transporting ATPase activity, rotational mechanism	SP_1510, SP_1508, SP_1509
GOTERM_CC_FAT	GO:0033177~proton-transporting two-sector ATPase complex, proton-transporting domain	SP_1512, SP_1513, SP_1514
GOTERM_BP_FAT	GO:0009165~nucleotide biosynthetic process	SP_1512, SP_1510, SP_1513, SP_1514, SP_1508, SP_1509
GOTERM_MF_FAT	GO:0019829~cation-transporting ATPase activity	SP_1510, SP_1508, SP_1509
GOTERM_BP_FAT	GO:0044271~nitrogen compound biosynthetic process	SP_0991, SP_1970, SP_1512, SP_1510, SP_1513, SP_2210, SP_1514, SP_1577, SP_1508, SP_1509

GOTERM_CC_FAT	GO:0033178~proton-transporting two-sector ATPase complex, catalytic domain	SP_1510, SP_1508, SP_1509
UP_SEQ_FEATURE	transmembrane region	SP_0013, SP_1512, SP_1513, SP_1514, SP_0799, SP_1882
GOTERM_BP_FAT	GO:0006812~cation transport	SP_1512, SP_1510, SP_1513, SP_1514, SP_1508, SP_1509
SP_PIR_KEYWORDS	transmembrane	SP_0877, SP_0013, SP_1512, SP_1461, SP_1778, SP_1513, SP_1514, SP_0799, SP_1882
GOTERM_MF_FAT	GO:0042625~ATPase activity, coupled to transmembrane movement of ions	SP_1510, SP_1508, SP_1509
GOTERM_BP_FAT	GO:0006811~ion transport	SP_1512, SP_1510, SP_1513, SP_1514, SP_1508, SP_1509
GOTERM_MF_FAT	GO:0016820~hydrolase activity, acting on acid anhydrides, catalyzing transmembrane movement of substances	SP_1510, SP_1580, SP_1508, SP_1509
GOTERM_CC_FAT	GO:0005886~plasma membrane	SP_0877, SP_0013, SP_1512, SP_1461, SP_1510, SP_1513, SP_1514, SP_0799, SP_1508, SP_1882
GOTERM_MF_FAT	GO:0042626~ATPase activity, coupled to transmembrane movement of substances	SP_1510, SP_1508, SP_1509
GOTERM_MF_FAT	GO:0043492~ATPase activity, coupled to movement of substances	SP_1510, SP_1508, SP_1509
GOTERM_MF_FAT	GO:0015399~primary active transmembrane transporter activity	SP_1510, SP_1508, SP_1509
GOTERM_MF_FAT	GO:0015405~P-P-bond-hydrolysis-driven transmembrane transporter activity	SP_1510, SP_1508, SP_1509
GOTERM_MF_FAT	GO:0042623~ATPase activity, coupled	SP_1510, SP_1508, SP_1509
GOTERM_MF_FAT	GO:0016887~ATPase activity	SP_1648, SP_1510, SP_1580, SP_1508, SP_1509
GOTERM_CC_FAT	GO:0016021~integral to membrane	SP_0877, SP_0013, SP_1512, SP_1461, SP_0283, SP_0626, SP_1778, SP_1513, SP_1514, SP_0799, SP_1882
GOTERM_CC_FAT	GO:0031224~intrinsic to membrane	SP_0877, SP_0013, SP_1512, SP_1461, SP_0283, SP_0626, SP_1778, SP_1513, SP_1514, SP_0799, SP_1882

Annotation Cluster 5	Enrichment Score: 1.3255425733230597	
Category	Term	Genes
GOTERM_MF_FAT	GO:0017076~purine nucleotide binding	SP_0786, SP_0724, SP_1982, SP_0820, SP_0600, SP_1219, SP_1653, SP_0338, SP_0720, SP_1523, SP_1717, SP_0516,

		SP_0519, SP_1276, SP_1051, SP_1749, SP_1087
SP_PIR_KEYWORDS	atp-binding	SP_0786, SP_0724, SP_1523, SP_1717, SP_0820, SP_0600, SP_1219, SP_1653, SP_1276, SP_0338, SP_1087, SP_0720
GOTERM_MF_FAT	GO:0001883~purine nucleoside binding	SP_0786, SP_0724, SP_1982, SP_0820, SP_0600, SP_1219, SP_1653, SP_0338, SP_0720, SP_1523, SP_1717, SP_0516, SP_0519, SP_1276, SP_1051, SP_1087
GOTERM_MF_FAT	GO:0030554~adenyl nucleotide binding	SP_0786, SP_0724, SP_1982, SP_0820, SP_0600, SP_1219, SP_1653, SP_0338, SP_0720, SP_1523, SP_1717, SP_0516, SP_0519, SP_1276, SP_1051, SP_1087
GOTERM_MF_FAT	GO:0001882~nucleoside binding	SP_0786, SP_0724, SP_1982, SP_0820, SP_0600, SP_1219, SP_1653, SP_0338, SP_0720, SP_1523, SP_1717, SP_0516, SP_0519, SP_1276, SP_1051, SP_1087
GOTERM_MF_FAT	GO:0032555~purine ribonucleotide binding	SP_0786, SP_1982, SP_0724, SP_1523, SP_1717, SP_0820, SP_0600, SP_1219, SP_0519, SP_1653, SP_1276, SP_1749, SP_0338, SP_1051, SP_1087, SP_0720
GOTERM_MF_FAT	GO:0032553~ribonucleotide binding	SP_0786, SP_1982, SP_0724, SP_1523, SP_1717, SP_0820, SP_0600, SP_1219, SP_0519, SP_1653, SP_1276, SP_1749, SP_0338, SP_1051, SP_1087, SP_0720
SP_PIR_KEYWORDS	nucleotide-binding	SP_0786, SP_0724, SP_1523, SP_1717, SP_0820, SP_0600, SP_1219, SP_1653, SP_1276, SP_0338, SP_1087, SP_0720
GOTERM_MF_FAT	GO:0000166~nucleotide binding	SP_0786, SP_0724, SP_1982, SP_0820, SP_0600, SP_1219, SP_1653, SP_0338, SP_0720, SP_1523, SP_1717, SP_0516, SP_0519, SP_1276, SP_1051, SP_1749, SP_1087
GOTERM_MF_FAT	GO:0032559~adenyl ribonucleotide binding	SP_0786, SP_1982, SP_0724, SP_1523, SP_1717, SP_0820, SP_0600, SP_1219, SP_0519, SP_1653, SP_1276, SP_0338, SP_1051, SP_1087, SP_0720
GOTERM_MF_FAT	GO:0005524~ATP binding	SP_0786, SP_1982, SP_0724, SP_1523, SP_1717, SP_0820, SP_0600, SP_1219, SP_0519, SP_1653, SP_1276, SP_0338, SP_1051, SP_1087, SP_0720
COG_ONTOLOGY	Defense mechanisms	SP_0786, SP_0600, SP_0509, SP_1653
INTERPRO	IPR003593:ATPase, AAA+ type, core	SP_0786, SP_1717, SP_0820, SP_0600, SP_1653, SP_0338, SP_0720
INTERPRO	IPR017871:ABC transporter, conserved site	SP_0786, SP_1717, SP_0600, SP_1653, SP_0720
SMART	SM00382:AAA	SP_0786, SP_1717, SP_0820, SP_0600, SP_1653, SP_0338, SP_0720

INTERPRO	IPR003439:ABC transporter-like	SP_0786, SP_1717, SP_0600, SP_1653, SP_0720
GOTERM_MF_FAT	GO:0016887~ATPase activity	SP_0786, SP_1717, SP_0600, SP_1653, SP_1087, SP_0720
