# Supplementary material for: Transcriptome analysis of Streptococcus pneumoniae treated with the designed antimicrobial peptides, DM3
Source: Sci Rep. 2016 May 26;6:26828. doi: 10.1038/srep26828 (PMC4881017; doi:10.1038/srep26828)
Supplement: Supplementary Information [file srep26828-s4.pdf]

Transcriptome analysis of *Streptococcus pneumoniae* treated with the designed antimicrobial peptides, DM3

Cheng-Foh Le, Ranganath Gudimella, Rozaimi Razali, Rishya Manikam & Shamala Devi Sekaran

Table S4. Pathway enrichment for PSSP comparing between with and without PEN treatment.

Annotation Cluster 5	Enrichment Score: 1.9164477693497612	
Category	Term	Genes
GOTERM_MF_FAT	GO:0004222~metalloendopeptidase activity	SP_0617, SP_2224, SP_2225
GOTERM_MF_FAT	GO:0004175~endopeptidase activity	SP_0617, SP_2224, SP_2225
GOTERM_MF_FAT	GO:0008237~metallopeptidase activity	SP_0617, SP_2224, SP_2225
GOTERM_MF_FAT	GO:0070011~peptidase activity, acting on L-amino acid peptides	SP_0617, SP_2224, SP_2225
GOTERM_BP_FAT	GO:0006508~proteolysis	SP_0617, SP_2224, SP_2225
GOTERM_MF_FAT	GO:0008233~peptidase activity	SP_0617, SP_2224, SP_2225
Cluster-1	No gene enrichment	
Cluster-2	No gene enrichment	
Cluster-3	No gene enrichment	
Cluster-4	No gene enrichment	
